# Outcome Measurement and Evaluation as a Routine practice in alcohol and other drug services in Belgium (OMER-BE)

**DOI:** 10.1192/j.eurpsy.2023.354

**Published:** 2023-07-19

**Authors:** C. Migchels, A. Zerrouk, F. Matthys, W. van den Brink, K. Fernandez, J. Antoine, W. Vanderplasschen, C. L. Crunelle

**Affiliations:** ^1^Department of Psychiatry, Vrije Universiteit Brussel (VUB), Universitair Ziekenhuis Brussel (UZ Brussel), Brussels; ^2^Department of Special Needs Education, Ghent University (UGent), Ghent, Belgium; ^3^Academic Medical Center, University of Amsterdam, Amsterdam, Netherlands; ^4^Department of Epidemiology and Public Health, Sciensano, Brussels, Belgium

## Abstract

**Introduction:**

There is a variety of specialized outpatient and residential Alcohol and Other Drugs (AOD) services, but research on outcomes of these services is limited. Given the chronic, relapsing nature of AOD problems, there is a need for longitudinal research on outcomes after treatment in various AOD services.

Patient-Reported Outcome Measures (PROMs) and Patient-Reported Experience Measures (PREMs) are hardly used in the AOD field but provide excellent tools and a framework to monitor progress and outcomes in these services based on experiences of service users.

**Objectives:**

The objectives of the OMER-BE study are to:

1. Assess and compare patient characteristics at baseline in various treatment modalities

2. Test and prepare the routine measurement of PROMs and PREMs in AOD services using a self-report tool

3. Assess patient-reported experiences qualitatively in various treatment modalities for AOD patients

The overall goal is to continuously assess and improve AOD services.

**Methods:**

We have set up a naturalistic, longitudinal cohort study for which we will engage and follow up 250 AOD users as they present themselves in selected AOD services in four different treatment modalities (outpatient non-pharmacological treatment, outpatient substitution treatment, residential psychiatric treatment and therapeutic communities for addictions).

Sociodemographic and clinical factors and PROMs will be assessed at baseline. PROMs and PREMs will be assessed at 45-, 90- and 180-days follow-up. The questionnaires that will be used during the baseline and follow-up assessments are based on the ICHOM Standard Set for Addictions (ICHOM SSA, 2020), a set of brief validated questionnaires to measure and monitor treatment outcomes routinely in AOD services.

Following the 6-month follow-up we will perform a qualitative study in a subset of N=20 participants (5 per treatment modality). These participants will be invited to take part in an in-depth interview with one of the researchers, where the following topics will be discussed: treatment history, recovery experiences, helping and hindering factors in recovery, and experiences with different treatment modalities.

**Results:**

47 participants have been recruited in the OMER-BE study up until October 2022. 38 (81%) of the participants were born male, 9 (19%) were born female. The average age of participants is 33, with ages ranging from 19 to 47 years old.

Preliminary findings will be presented at the congress.

Further recruitment of study participants up to a total of 250 will be undertaken until the end of 2023.

**Conclusions:**

The OMER-BE study aims to assess and improve AOD services by using a self-report tool, measuring PROMs and PREMs.

**Disclosure of Interest:**

None Declared

